# Penile skin necrosis: A case report of a rare complication associated with simultaneous circumcision utilizing a disposable circumcision suture device and genital beading surgery

**DOI:** 10.1097/MD.0000000000042355

**Published:** 2025-05-02

**Authors:** Bing-Tau Chen, Chien-Ming Lai, Chiao-Ching Li, Ying-Jui Ni, Chiang-Ting Wang, Chun-Feng Chang

**Affiliations:** a Division of Urology, Department of Surgery, Kaohsiung Armed Forces General Hospital, Kaohsiung City, Taiwan; b Division of Urology, Department of Surgery, Tri-Service General Hospital, National Defense Medical Center, Taipei, Taiwan.

**Keywords:** circumcision, complication, disposable circumcision suture device, skin necrosis

## Abstract

**Rationale:**

Male circumcision is a widely performed procedure globally and is generally safe. However, penile skin necrosis is a rare but serious complication. To our knowledge, this is the first case report describing the management of complications arising from circumcision using a disposable circumcision suture device (DCSD) performed simultaneously with a genital beading procedure.

**Patient concerns:**

A healthy 37-year-old man underwent circumcision using a DCSD and a genital beading procedure by an unlicensed practitioner.

**Diagnoses:**

Wound infection with skin necrosis over the penile shaft.

**Interventions and outcomes:**

The patient required surgical debridement and single-stage reconstruction with a local flap. After appropriate surgical intervention and antibiotic treatment, the wound healed well. No structural or functional abnormalities were noted during outpatient follow-up. Ethical approval was waived due to case report only. Informed consent was given.

**Lessons:**

Although the DCSD offers benefits such as reduced operative time and improved cosmetic outcomes compared with conventional circumcision, combining the procedure with genital beading is not recommended due to the increased risk of complications. There are many techniques for reconstructing penile skin loss including split-thickness skin grafts, spiral flap, rotational skin flaps, pedicled skin grafting with vascular anastomosis, etc. Complex reconstruction is not always necessary; single-stage reconstruction with a local flap is a relatively simple but effective approach in certain situations.

## 1. Introduction

In Western Europe, North America, and the Middle East, male circumcision is predominantly performed on children or infants for religious and cultural reasons.^[1–3]^ By contrast, circumcision is less common in East Asia, although it remains widely practiced in Taiwan.^[3]^

Male circumcision is one of the most frequently performed surgical procedures, with a tradition spanning millennia.^[4]^ Approximately 16.7% to 30% of the global male population has undergone circumcision.^[5–7]^

Recent research indicates that the disposable circumcision suture device (DCSD) is efficient and safe,^[8–11]^ a finding that explains its increasingly widespread use. Studies have reported that the infection rate following circumcision ranges from 0.4% to 1.3%,^[8,12,13]^ with skin necrosis being a rare but serious complication.

## 2. Case report

A healthy 37-year-old man underwent circumcision using a DCSD along with a simultaneous genital beading procedure by an unlicensed practitioner. Postoperatively, he experienced severe penile swelling and pain. Despite seeking treatment at a local clinic, his symptoms persisted. He subsequently presented to our emergency department with wound dehiscence and skin necrosis.

**Figure 1.**
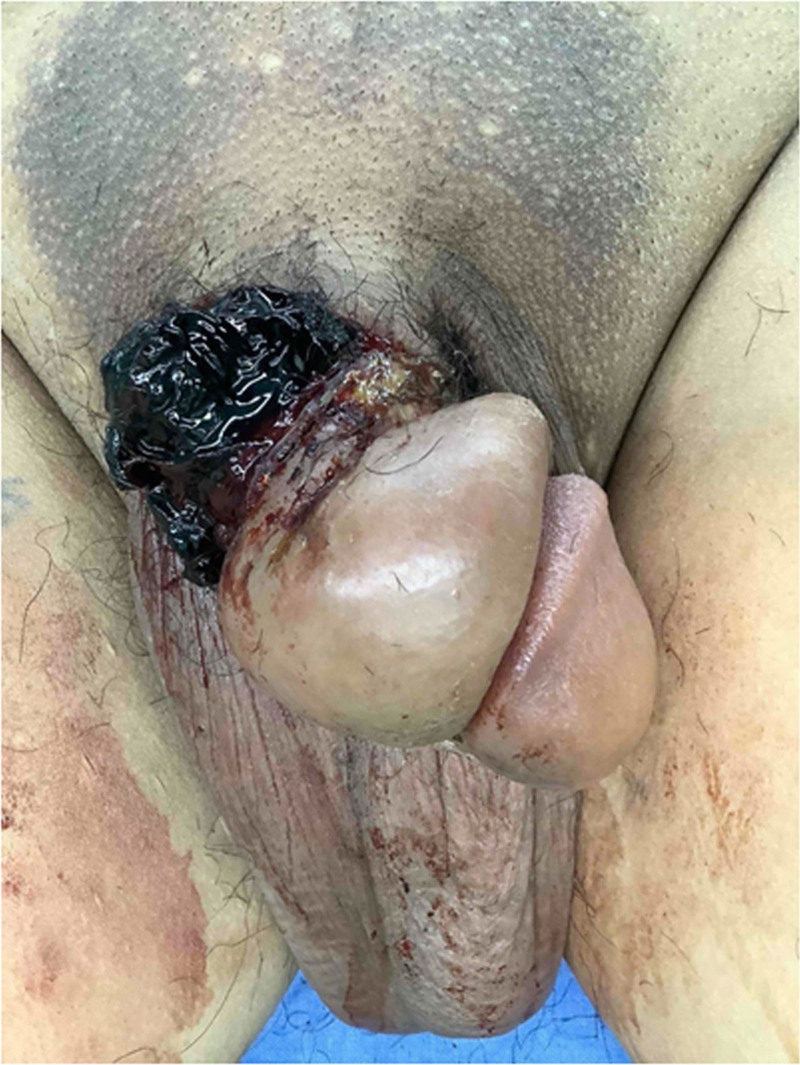
Wound dehiscence and skin necrosis after circumcision, with the wound located at the base of the penile shaft.

**Figure 2.**
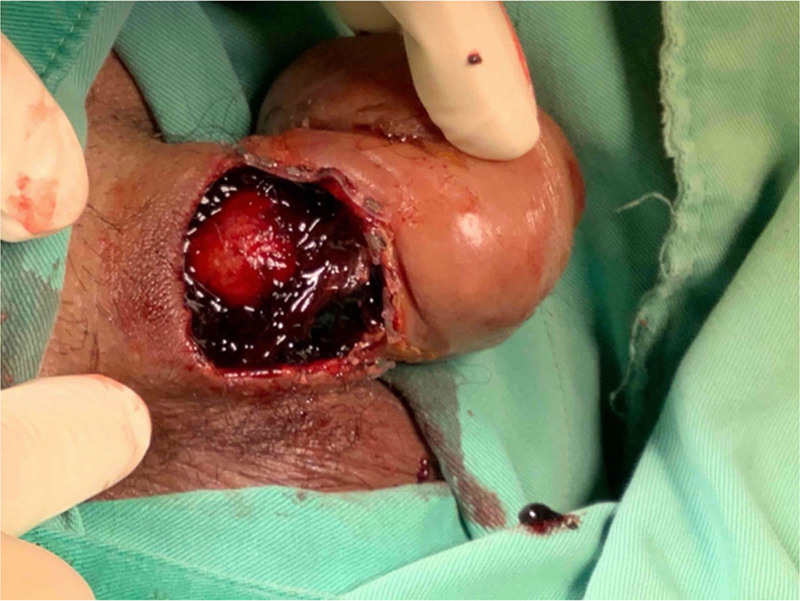
After the necrotic tissue was removed, multiple blood clots were observed.

Examination revealed that the circumcision wound was located at the base of the penile shaft rather than at the typical distal site near the glans sulcus (Fig. 1). The patient presented with severe soft tissue edema and inadequate perfusion of the subcutaneous layer of the prepuce. The wound was characterized by dehiscence, extensive necrosis, and multiple blood clots (Fig. 2).

**Figure 3.**
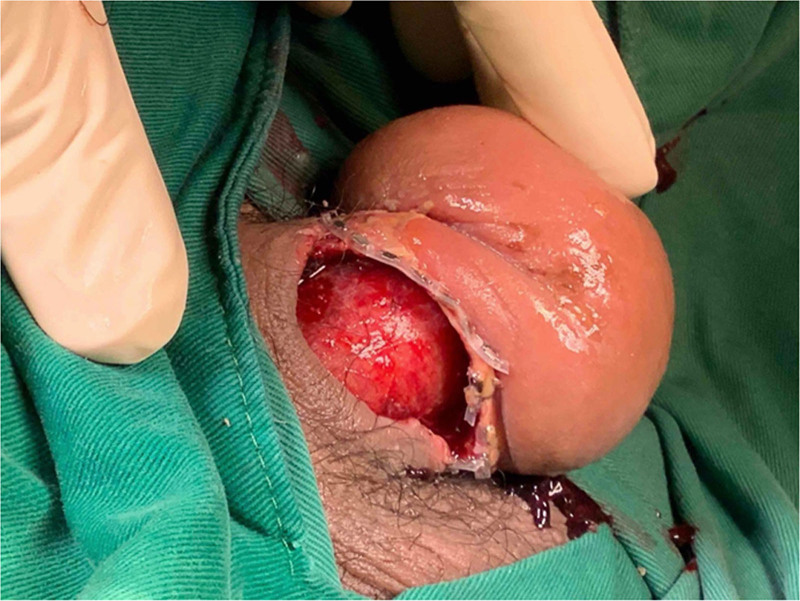
No active bleeding was noted, and the tunica albuginea was intact.

**Figure 4.**
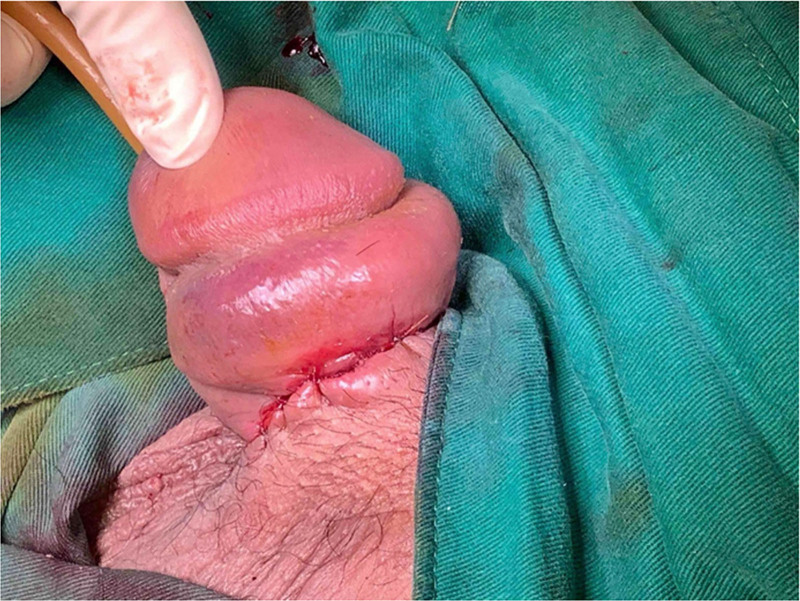
Sufficient soft tissue remained for tension-free primary closure.

**Figure 5.**
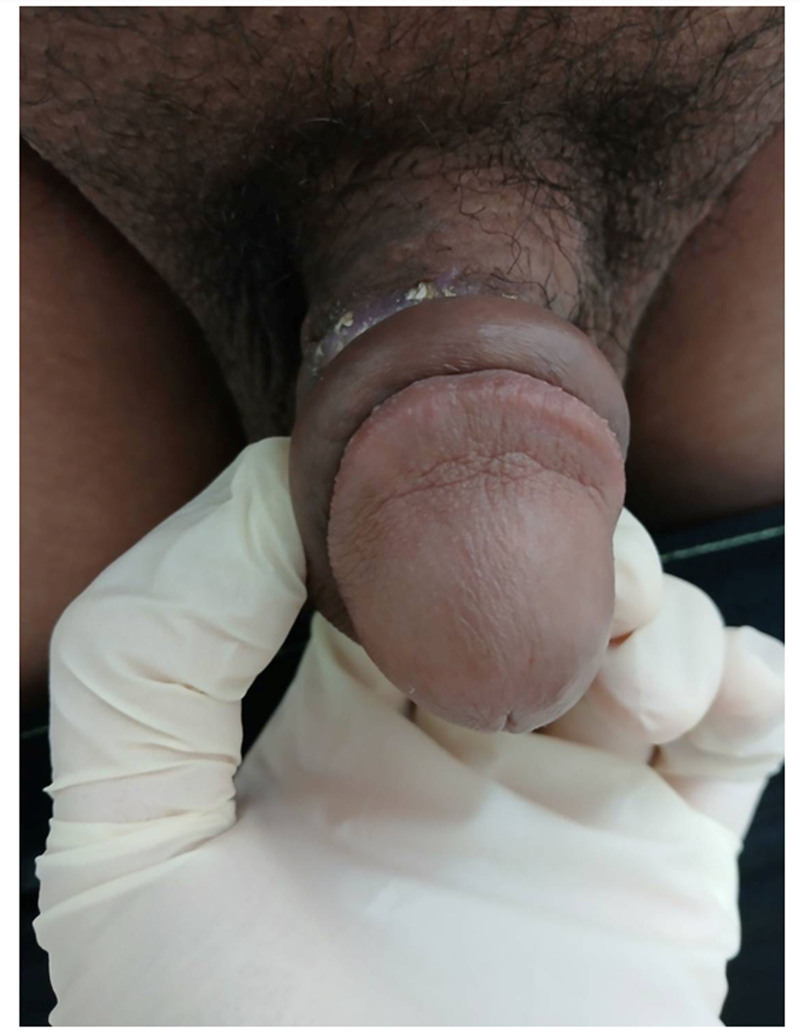
The wound healed well; the genital beading involved an elastic circular band implanted near the coronal sulcus.

After admission, a debridement was performed to manage the wound utilizing excess tension-free soft tissue, and single-stage reconstruction with a local flap was achieved (Figs. 3 and 4). With appropriate wound care and antibiotics, the wound healed well, and the patient was discharged. Once the edema subsided, we discovered that his genital beading consisted of an elastic circular band implanted near the coronal sulcus (Fig. 5). The patient was satisfied with the outcome of the treatment. Informed consent was obtained from the patient for publication of this case report details.

## 3. Discussion

### 3.1. Conventional circumcision and complications

In East Asia, including Taiwan, men or boys undergo circumcision at various ages due to conditions such as phimosis and balanitis, hygiene concerns, or cosmetic preferences.^[3,6,14,15]^ Several circumcision methods are available, with the most common for adults being the forceps-guided, dorsal slit, and sleeve resection methods.^[4,6,16]^ These procedures are not associated with an increased risk of complications.^[2]^

The forceps-guided method, introduced by Sir Frederick Treves in 1903,^[4]^ involves placing long straight forceps across the foreskin from the 6 o’clock to 12 o’clock positions, followed by excision with a scalpel.^[16]^ The dorsal slit technique, which requires greater surgical expertise, is typically recommended for patients with phimosis or paraphimosis.^[6,16]^ By contrast, the sleeve resection method, which demands skilled surgical assistance, involves 2 incisions to remove an appropriate amount of skin and generally results in superior cosmetic outcomes compared with the other techniques.^[6,16]^

Numerous studies have indicated that most complications associated with circumcision are minor, with infection, bleeding, and meatal stenosis being the most common.^[1,2,12]^ A systematic review conducted in 2021 estimated an overall complication rate of 4%, revealing that therapeutic circumcisions had more than double the complication rate of nontherapeutic circumcisions (7.47%; 95% confidence interval [CI]: 5.40–9.84 vs. 3.34%; 95% CI: 2.86–3.85).^[2]^

In most cases, postcircumcision bleeding is effectively managed with direct pressure or silver nitrate, and wound exploration and suturing are rarely required.^[12,17,18]^ The penis is supplied with blood from the superficial external pudendal artery and the internal pudendal artery, which connect at the coronal sulcus.^[19]^ This dual supply explains the rarity of postcircumcision wound infections.^[11]^

Skin necrosis is a rare but serious complication of circumcision. Gul et al^[20]^ reported 2 cases of necrosis following the administration of local anesthesia containing epinephrine. Additionally, İnce et al^[21]^ reported a case of necrotic penile skin successfully treated with primary suturing.^[21]^

### 3.2. DCSD versus conventional circumcision

The DCSD is a commercially designed circular stapler^[8]^ that uses circular blades to excise the foreskin followed by a continuous suture along the cut edge to close the tissue.^[8,10]^ The device functions similarly to a stapling sewing machine.^[10]^ Complication rates for the DCSD vary across studies, with common problems including edema, hematoma, infection, and instrument malfunction.^[9,11]^ Multiple studies have suggested that the DCSD is safer and more efficient than conventional circumcision (CC).^[8,9,22]^

A systematic review of 9 randomized controlled trials involving 1898 patients revealed no statistically significant differences in dehiscence or hematoma rates between patients treated with CC and the DCSD. However, the DCSD possesses several advantages over traditional methods, such as shorter surgical times (−21.44 minutes; 95% CI:−25.08 to −17.79), faster wound healing (−3.66 days; 95% CI: −5.46 to −1.85), less intraoperative bleeding (−9.64 mL; 95% CI: −11.37 to −7.90), and superior cosmetic outcomes (8.77; 95% CI: 5.90–13.02). Additionally, patients reported lower pain levels during the procedure and within the first 24 hours following surgery, in addition to reduced infection risk, incision edema, and overall complications.^[10]^

A retrospective analysis of 394 cases in Taiwan highlighted the requirement for caution when using the DCSD on older patients, those who are obese, or those with phimosis and diabetes mellitus (DM) because these patients experience increased risks of infection, dehiscence, and hematoma. Despite these risks, the DCSD is a safe and effective alternative to CC.^[11]^

### 3.3. Genital beading/pearling

Subcutaneous penile modification, commonly referred to as genital beading or peeling, is widespread in Southeast Asia and East Asia.^[23]^ The practice is also increasingly common in Europe and the United States.^[24]^ Motivations for undergoing genital beading include enhancing sexual pleasure, fulfilling a partner’s request, or expressing one’s masculinity.^[25,26]^

Genital beading is often performed outside of medical institutions under nonsterile conditions and involves making small incisions on the penile shaft. Foreign materials such as glass, metal, nylon, silicone, or plastic are inserted into the subcutaneous tissue using a rod.^[24–26]^ Complications associated with genital beading comprise infection, pain or trauma during sexual intercourse, difficulties using condoms, and implant extrusion.^[24–28]^

### 3.4. What accounts for skin necrosis?

After circumcision, the remaining preputial skin receives its blood supply from the Dartos fascia, maintained by retrograde flow from the glans.^[19]^ In the case of the patient in the present study, the genital beading occurred near the glans. To mitigate the presence of a foreign body, the practitioner likely situated the circumcision incision at the base of the penile shaft. During the procedure, the elastic, mobile skin may be elevated from the shaft to prevent damage to the underlying tunica albuginea and erectile tissues.^[25]^ However, the subcutaneous tissue, including the Dartos fascia, may sustain injury during the procedure, as occurred in the patient in this study.

Postoperative edema can compress tissues, potentially reducing blood flow and leading to inadequate perfusion, which may cause skin necrosis. Additionally, the use of epinephrine in the anesthesia can exacerbate this condition.

### 3.5. Reconstruction of penile skin defects

Penile reconstruction requires careful consideration of the unique anatomical features of the penis. The penile skin is nonhair-bearing and thin, easily stretches over the underlying tissues, and must maintain sufficient elasticity to accommodate expansion during sexual stimulation.^[29]^

Several techniques for penile skin reconstruction have been developed. Some involve rotational skin flaps from areas with excess tissue, such as the scrotum^[30–32]^ or the pudendal thigh flap.^[33]^ Alternatively, full-thickness skin grafts can be harvested from the thigh, scrotum, or inguinal region.^[19,34,35]^

In the case of the patient in the present study, sufficient soft tissue facilitated a tension-free primary closure. Patients must be closely monitored following reconstruction because several factors can contribute to graft failure, including obesity, DM, hyperglycemia, poor nutrition, and an infected or colonized wound bed. Managing these factors is crucial to successful graft integration.^[19]^

### 3.6. Limitations

The limitation of this study is that it is a single case report. Patients who undergo both circumcision and genital beading simultaneously are rare, making it difficult to conduct a randomized controlled study. Therefore, this study serves only as a report sharing experiences in the management of surgical complications.

## 4. Conclusion

Circumcision is a widely performed procedure worldwide, but it must be performed by a qualified, licensed surgeon with proper training. Genital beading is discouraged due to its inherent risks and potential complications, particularly when performed concurrently with circumcision, which increases the risk of adverse outcomes. Reconstructing penile skin is challenging, and selecting an appropriate reconstruction method is crucial to improving success rates, patient satisfaction, and cosmetic outcomes.

## Acknowledgments

The author wishes to acknowledge the help of Dr. Chang in data collection and Dr Ni in commenting on an early draft of the chapter.

## Author contributions

**Conceptualization:** Bing-Tau Chen, Chiao-Ching Li, Chiang-Ting Wang.

**Data curation:** Bing-Tau Chen.

**Formal analysis:** Bing-Tau Chen, Ying-Jui Ni.

**Writing – original draft:** Bing-Tau Chen.

**Writing – review & editing:** Bing-Tau Chen, Ying-Jui Ni, Chun-Feng Chang.

**Software:** Chien-Ming Lai.

**Resources:** Chun-Feng Chang.
